# Lamotrigine, quetiapine and aripiprazole-induced neuroleptic malignant syndrome in a patient with renal failure caused by lithium: a case report

**DOI:** 10.1186/s12888-020-02597-x

**Published:** 2020-04-19

**Authors:** Anna Maria Szota, Izabela Radajewska, Przemysław Grudzka, Aleksander Araszkiewicz

**Affiliations:** Department of Psychiatry, Collegium Medicum in Bydgoszcz, Nicolaus Copernicus University in Toruń, 9 Curie-Skłodowskiej Street, 85-094 Bydgoszcz, Poland

**Keywords:** Neuroleptic malignant syndrome, Lamotrigine, Quetiapine, Aripiprazole, Lithium

## Abstract

**Background:**

Neuroleptic malignant syndrome (NMS) may be induced by atypical antipsychotic drugs (AAPDs) such as aripiprazole, olanzapine, risperidone and quetiapine, either as a single treatment or in combination with other drugs. A case of NMS following the administration of lamotrigine, aripiprazole and quetiapine in a patient with bipolar disorder, and with renal failure caused by toxic lithium levels has not been reported.

**Case presentation:**

A 51-year-old female patient with a 27-year history of bipolar disorder, being treated with lithium, fluoxetine, olanzapine, gabapentine, perazine and biperiden, was admitted to the hospital due to depressed mood and delusions. A urinary tract infection was diagnosed and antibiotic therapy was initiated. After 5 days of treatment her physical state deteriorated and she developed a fever of 38.4 °C. Her laboratory results revealed a toxic level of lithium (2.34 mmol/l). Acute renal failure was diagnosed and the lithium was withdrawn. After stabilization of her condition, and despite her antipsychotic treatment, further intensification of delusions and depressed mood were observed. All drugs being taken by the patient were withdrawn and lamotrigine and aripiprazole were initiated. Due to the insufficient effectiveness of aripiprazole treatment and because of problems with sleep, quetiapine was added, however further treatment with this drug combination and an increase of quetiapine to 400 mg/d eventually caused NMS. Amantadine, lorazepam and bromocriptine were therefore initiated and the patient’s condition improved.

**Conclusion:**

This case report indicates that concurrent use of multiple antipsychotic drugs in combination with mood stabilizers in patients with organic disorders confers an increased risk of NMS development.

## Background

Neuroleptic malignant syndrome (NMS) is a rare, severe and potentially fatal condition characterized by hyperthermia (> 38 °C), muscle rigidity, changes in mental status (delirium, altered consciousness), increased activity of the autonomic nervous system (tachycardia, diaphoresis, blood pressure elevation), increased levels of creatine phosphokinase (CPK) and leukocytosis [1]. There are many risk factors responsible for NMS development such as the use of high-dose, high-potency and long-acting antipsychotic drugs (APDs), a rapid increase in the dosage of an APD, using multiple APDs or other medications like antidepressants and mood stabilizers, especially lithium. Previous NMS, dehydration, physical exhaustion, renal failure, a family history of catatonic syndrome and dementia can also put the patient at risk [2–4]. NMS is caused mainly by typical antipsychotic drugs (TAPDs), but reports of AAPD (aripiprazole, quetiapine, olanzapine or risperidone) induced NMS are well documented [5]. So far, only two cases of NMS induced by lamotrigine and antipsychotics such as aripiprazole and risperidone have been reported [6, 7]. However, in these cases additional risk factors (infectious disease, renal failure, dehydration) which have an influence on NMS development were excluded [6, 7].

We present here a case of lamotrigine, quetiapine and aripiprazole-induced neuroleptic malignant syndrome in a patient with renal failure caused by lithium. It is worth pointing out that a case such as this has not yet been reported. The purpose of this case report is to increase awareness and vigilance of the fact that concurrent use of multiple antipsychotic drugs in combination with mood stabilizers in patients with somatic disease may significantly increase the risk of NMS development.

## Case presentation

A 51-year-old female patient with a 27-year history of bipolar disorder was admitted to the psychiatric clinic in September 2017 due to depressed mood, ideas of reference, delusions of persecution and anxiety. It was known from her medical history that her first psychotic episode (acute paranoid syndrome) occurred in 1990. The patient was hospitalized for a few weeks and was treated with antipsychotic drug (perazine) with a good result. Within the next 10 years the patient was hospitalized 4 times and diagnosed with depression and mania. The depressive episodes were treated with different antidepressant drugs (imipramine, perazine and venlafaxine) in combination with mood stabilizers (carbamazepine and/or lithium), whereas antipsychotic drugs were used to treat the mania. After the last hospitalization (in 2000) for depression, the patient continued her treatment (imipramine, perazine, lithium and carbamazepine) as an out-patient until 2008. The treatment of the patient was modified due to fluctuations in her mental state. Imipramine and carbamazepine were withdrawn and fluoxetine was commenced. In 2015, the patient was hospitalized owning to acute intoxication with lithium (> 1.2 mmol/l), renal failure and extrapyramidal symptoms. Lithium was withdrawn and gabapentine was initiated for the extrapyramidal symptoms. In addition, hypothyroidism was diagnosed and levothyroxine was prescribed, which patient took until 2016. Within the next 2 years the patient was taking fluoxetine (60 mg/d), perazine (150 mg/d) and gabapentine (800 mg/d). During that time carbamazepine was commenced (200 mg/d, max. to 800 mg/d) to stabilize the patient’s mood, but owing to severe deterioration of her mental state (intensification of both psychotic and depressive symptoms), the drug was withdrawn and lithium (max.1000 mg/d) was reintroduced. Additionally, olanzapine (5 mg/d) for psychotic symptoms and biperiden (4 mg/d) for muscle stiffness were added. Throughout the disease (since the first psychotic episode in 1990 until the last hospitalization in 2017), there were no psychiatric comorbidities diagnosed. Periodic hypothyroidism (in 2015) treated with levothyroxine and renal failure caused by acute intoxication with lithium were notable for the patient’s history.

The patient was admitted to the psychiatric clinic in September 2017 due to depressed mood, ideas of reference, delusions of persecution and anxiety. During her admission disturbances of consciousness were not observed and the patient was auto- and allopsychically orientated. The patient’s general condition was good (blood pressure: 115/70 mmHg, pulse 72, respiratory rate16/min and BMI 27,22). A regular systolic murmur was noted and lungs were clear bilaterally. The abdomen was symmetrical without distention and bowel sounds were normal in quality and intensity in all areas. The patient had dry skin and varicose veins of the lower extremities. Her laboratory results showed lithium levels of 1.48 mmol/l, GFR 35 ml/min, creatinine 1.56 mg/dl and WBC 10.42 × 10^3/ml. A urinary tract infection was diagnosed (leukocytosis 10.2 × 10^3/ml, positive result of urine inoculation-Klebsiella oxytoca) and antibiotic therapy was initiated (sulfamethoxazole with trimethoprim). After 5 days of treatment her physical state deteriorated, she developed a fever of 38.4 °C and her laboratory results revealed a toxic level of lithium carbonate 2.34 mmol/l, creatinine 2.05 mg/dl and GFR 25 ml/min. Acute renal failure was diagnosed by a nephrologist and as a result the lithium was withdrawn and the antibiotic therapy was replaced with ceftriaxone 2 g/d (lower nephrotoxicity). Normal saline was administered to the patient for two days in order to decrease the toxic level of lithium. The patient’s general condition and laboratory findings improved (level of lithium 1.34 mmol/l, creatinine 1.94 mg/l, GFR 27 ml/min), and 7 days later the antibiotic therapy was discontinued. Other laboratory results (biochemistry, hematology, lipidemia, ions and hormone levels- TSH, fT3, fT4) were found to be within normal range, so other somatic disorders, apart from renal failure, were excluded.

After stabilization of her somatic condition, and despite her treatment (the patient was continuing treatment with fluoxetine 60 mg/d, olanzapine 5 mg/d, gabapentin 800 mg/d, perazine 150 mg/d and biperiden 4 mg/d), the patient’s mental state deteriorated significantly (depressed mood, ideas of reference, aggravated delusions of persecution), so the treatment was changed accordingly. All drugs taken by the patient so far were gradually withdrawn (olanzapine and perazine over 6 days; fluoxetine over 4 weeks, gabapentine over 3 weeks and biperiden over 1 week) and lamotrigine (200 mg/d) and aripiprazole (30 mg/d) were initiated. Lamotrigine was used to replace lithium, as it is a good mood stabilizer without any nephrotoxic influence on the kidneys. Lamotrigine was commenced at a dose of 25 mg/d, which the patient took for 2 weeks. The dosage was then increased to 50 mg/d for a week, and then incrementally increased each week up to the final dose of 200 mg/d. Aripiprazole was initiated at a dose of 7.5 mg/d in order to reduce delusions and was uptitrated to the final dose of 30 mg/d over 2 weeks. As a result of the insufficient effectiveness of aripiprazole and lamotrigine treatment, which lasted 6 weeks, (ideas of reference, delusions of persecutions were maintained and problems with sleep), quetiapine was added (50 mg/d) in the 6th week. A partial good response to the treatment was observed after 7 days (partial reduction of delusions and mood stabilization), so the doctors decided to uptitrate the quetiapine dose to 400 mg/d, which took 9 days. Further treatment with aripirazole, lamotrigine and quetiapine resulted in NMS development. A description of NMS and its treatment is presented in Table 1.

**Table 1:** Symptoms of NMS and treatment

1.Symptoms of NMS	sudden agitation, delusional beliefs; consciousness disturbances; fever over 39 °C.; ‘cogwheel ‘muscle rigidity; tremor and sopor; vital signs as follows; blood pressure 80/50 mmHg and heart rate 100 beats/minute.
2. Laboratory results	CRP 334,90 mg/dl, LDH 357 U/l, CPK 6988 U/I, GFR 20 ml/min, creatinine 2.55 mg/dl.
3. NMS treatment	a. All antipsychotic drugs (aripiprazole and quetiapine) and lamotrigine were withdrawn. b. Amantadine (up to 200 mg/d), lorazepam (3 mg/d) and bromocriptine (3.75 mg/d) were initiated. c. The patient’s condition improved after 5 days of the treatment.
4.Further treatment of the patient	a. Because of severe psychotic symptoms clozapine and lamotrigine were commenced very slowly. Clozapine has a low risk of causing NMS and was titrated from 12.5 mg/per week to a dose of 200 mg/d, whereas lamotrigine was titrated from 25 mg/d every two weeks to a dose of 225 mg/d. b. After 4 months of treatment, clinical improvement was observed and the patient was discharged from hospital in a good general condition.

*CPK* creatine phosphokinase, *CRP* C reactive protein, *GFR* glomerular filtration rate, *LDH* lactate dehydrogenase, *NMS* neuroleptic malignant syndrome

To assess and quantify the causal role of antipsychotic drugs in the mediation of NMS, the Adverse Drug Reaction (ADR) Probability Scale [8] was used. According to this scale each drug was assigned to a probability category on the basis of points, which were calculated as follows: aripiprazole 2, lamotrigine 3 and quetiapine 5. Number of points: > 9 means definitive adverse drug reaction; 5 to 8 points- probable adverse drug reaction; 1 to 4- possible adverse drug reaction and 0- doubtful adverse drug reaction [8].

## Discussion and conclusions

To our knowledge, this is the first reported case of NMS induced by a combination of lamotrigine, aripiprazole and quetiapine in a patient with renal failure induced by toxic levels of lithium. Development of NMS in this patient was the result of the accumulation of a few different risk factors, the treatment with lithium being one of them [9]. As it is known from previous case reports, co-administration of lithium with antipsychotic medication [10] and the coexistence of renal failure and dehydration, as a result of the nephrotoxic influence of lithium, [11] increases the risk of NMS development. Even a toxic level of lithium per se might be responsible for NMS development [12–14]. In our patient long-term, controlled treatment with lithium resulted in renal failure, which intensified when a toxic level of lithium (> 1.2 mmol/l) was reached. As a result, the patient was hospitalized (July 2015). Lithium was gradually withdrawn in order to improve renals’ function and carbamazepine was initiated (200 mg/d; max. to 600 mg/d). Carbamazepine is a good mood stabilizer which may be used to prevent relapses of affective bipolar disorders, if there are contraindications to lithium [15]. Unfortunately, when carbamazepine was commenced in this case, both psychotic and depressive symptoms intensified, so carbamazepine was withdrawn and lithium was reintroduced. The level of lithium was regularly monitored (within the reference values) until the last psychiatric hospitalization in September 2017 when it exceeded the toxic value (2.34 mmol/l). This was probably associated with an inflammatory state in the kidneys caused by the bacteria Klebsiella oxytoca, and acute renal insufficiency development. An inflammatory state combined with fever and dehydration, also belong to systemic factors which can increase the likelihood of NMS development [16]. Therefore, renal failure- induced by lithium, dehydration, the inflammatory state and fever, in combination with another risk factor (pharmacotherapy), were responsible for the onset of NMS.

What is more, simultaneous treatment with aripiprazole and quetiapine [17, 18], which can increase the risk of NMS by partially blocking the dopaminergic system, and increased quetiapine dosage, due to the intensification of psychosis, also had an influence on the development of NMS in our patient [2]. The contribution of aripiprazole and quetiapine in our patient’s development of NMS was evaluated by using the Naranjo Adverse Drug Reaction (ADR) Probability Scale [8], which revealed the possible influence of aripiprazole and the probable influence of quetiapine in NMS development. Simultaneously, the patient was taking lamotrigine, which was commenced instead of the nephrotoxic lithium, to stabilize her mood. Lamotrigine is a well-tolerated drug but with common side effects such as fatigue, drowsiness, dizziness, headaches and rash [19], which were not observed in our patient. According to the Naranjo Adverse Drug Reaction (ADR) Probability Scale [8], the involvement of lamotrigine in NMS development was evaluated as possible. On the basis of the ADR Probability Scale we may conclude that both aripiprazole and lamotrigine had a similar and possible impact on NMS development, whereas quetiapine, the dose of which was uptitrated during the treatment, also had a probable influence on NMS development. Therefore, it may be presumed that quetiapine had a higher impact on NMS development than aripirazole and lamotrigine. However, it is worth noting, that all these 3 drugs (aripiprazole, lamotrigine and quetiapine) were taken by the patient at the same time and uptitration of the quetiapine dose took 9 days. So, the suggestion that only uptitration of the quetiapine dose, without taking into consideration aripiprazole and lamotrigine, being a direct cause of NMS development is not correct. Moreover, other risk factors of NMS development (renal failure induced by lithium, inflammation, dehydration and fever) were identified in the patient, which indicates multifactorial etiology of this case of NMS.

Although it has been proven that there is no pharmacokinetic interaction between lamotrigine and antipsychotic medication [20], lamotrigine may have an influence on NMS development by a different mechanism. Lamotrigine does not directly affect the dopaminergic system [21], but instead blocks sodium channels, and inhibits the release of glutamate along with inhibiting γ-aminobutyric acid (GABA) release [22]. It is hypothesized that the GABA-associated properties of lamotrigine may contribute towards NMS development [7]. The exact mechanism for how NMS is associated with the GABAergic system is not yet completely understood, but it is known that using GABAergic medication e.g. baclofen, may induce typical symptoms of NMS [23]. So far, only two cases of NMS induced by lamotrigine and antipsychotics have been reported [6, 7], however neither cases reported concomitant risk factors of NMS, as it is in our patient’s case.

When NMS was diagnosed in our patient all drugs (aripiprazole, quetiapine and lamotrigine) were withdrawn at once and amantadine (up to 200 mg/d), lorazepam (3 mg/d) and bromocriptine (3.75 mg/d) were commenced. This treatment was effective and 5 days later symptoms of NMS disappeared. However, psychotic symptoms (delusions of reference and delusions of persecution) and mood disturbances (agitation and irritability) significantly aggravated, so clozapine (from a dose of 12.5 mg/per week to 200 mg/d) and lamotrigine (from a dose of 25 mg/d each 2 weeks to 225 mg/d) were very slowly initiated. Clozapine, apart from its antipsychotic influence, does not cause extrapyramidal symptoms and has a low potential to cause NMS [24]. Lamotrigine was very slowly reintroduced as the patient’s mood was unstable. Using other drugs such as lithium or carbamazepine was not recommended due to chronic renal failure or intensification of psychotic symptoms, respectively. Moreover, in the doctors’ opinion, reintroduction of lamotrigine after NMS was justified by the fact that NMS was not induced by lamotrigine per se and lamotrigine was used in combination with another drug (clozapine), not in combination with drugs which caused NMS, namely aripiprazole and quetiapine.

Our knowledge of NMS-induced by lamotrigine and antipsychotic drugs is still very limited, mainly due to such a small number of published case reports. It may follow from the fact that medical help to some patients with NMS may be provided in the emergency department and in some cases NMS may be fatal for patients. Such cases may be less likely to be reported. As a consequence, a reliable estimation of the relative risk of NMS due to treatment with lamotrigine and antipsychotics is an open matter.

To sum up, the case presented here shows that treatment with a combination of a few drugs: aripiprazole, quetiapine and lamotrigine in patients with renal failure caused by lithium may cause NMS. This is why doctors in their everyday practice should be more aware of the fact that concurrent use of multiple antipsychotic drugs in combination with mood stabilizers in patients with somatic diseases may significantly increase the risk of NMS development. Therefore, early detection and appropriate treatment when NMS development is suspected may prevent death.

## Data Availability

The datasets generated during and/or analyzed during the current study are not publicly available due to law restrictions and hospital regulations which strictly limit who can access the data, but are available from the corresponding author on reasonable request.

## Abbreviations

AAPD: Atypical antipsychotic drugs
CPK: Creatine phosphokinase
CRP: C reactive protein
fT3: free triiodothyronine
fT4: free thyroxine
GABA: γ-aminobutyric acid
GFR: Glomerular filtration rate
LDH: Lactate dehydrogenase
NMS: Neuroleptic malignant syndrome
TAPD: Typical antipsychotic drugs
TSH: Thyroid stimulating hormone
WBC: White blood corpuscles
